# Pose Estimation for Straight Wing Aircraft Based on Consistent Line Clustering and Planes Intersection

**DOI:** 10.3390/s19020342

**Published:** 2019-01-16

**Authors:** Xichao Teng, Qifeng Yu, Jing Luo, Xiaohu Zhang, Gang Wang

**Affiliations:** 1 College of Aerospace Science and Engineering, National University of Defense Technology, Changsha 410073, China; yuqifeng@vip.sina.com (Q.Y.); zhangxiaohu@nudt.edu.cn (X.Z.); wanggang_nudt@163.com (G.W.); 2 High-Tech Institute, Qing Zhou 262500, China; luoj11@tsinghua.org.cn; 3 School of Aeronautics and Astronautics, Sun Yat-Sen University, Guangzhou 510000, China

**Keywords:** pose estimation, straight wing aircraft, structure extraction, consistent line clustering, parallel line, planes intersection

## Abstract

Aircraft pose estimation is a necessary technology in aerospace applications, and accurate pose parameters are the foundation for many aerospace tasks. In this paper, we propose a novel pose estimation method for straight wing aircraft without relying on 3D models or other datasets, and two widely separated cameras are used to acquire the pose information. Because of the large baseline and long-distance imaging, feature point matching is difficult and inaccurate in this configuration. In our method, line features are extracted to describe the structure of straight wing aircraft in images, and pose estimation is performed based on the common geometry constraints of straight wing aircraft. The spatial and length consistency of the line features is used to exclude irrelevant line segments belonging to the background or other parts of the aircraft, and density-based parallel line clustering is utilized to extract the aircraft’s main structure. After identifying the orientation of the fuselage and wings in images, planes intersection is used to estimate the 3D localization and attitude of the aircraft. Experimental results show that our method estimates the aircraft pose accurately and robustly.

## 1. Introduction

Since the 3D pose parameters of aircraft could provide a lot of valuable information about the aircraft’s flight status, effective and accurate pose estimation is a key technique in many aerospace applications, such as autonomous navigation [1], auxiliary landing [2], collision avoidance [3], accident analysis, and testing of a flight control system [4,5]. In recent years, with the development of imaging technology and computer vision, vision-based pose estimation has become a research hotspot, and a lot of methods have been proposed in the literature to estimate the pose of an aircraft using visual sensors. Visual sensors could be successfully applied in aircraft pose estimation since vision-based methods have the advantages of strong anti-interference ability, low cost, and high precision [6].

Vision-based pose estimation methods can be divided into two categories—on-board vision and external vision—depending on the mounting position of the visual sensors. On-board monocular, depth, or stereo cameras can be used in on-board vision methods to estimate the relative pose between the aircraft and a particular target or marker, while external vision methods utilize external cameras to acquire the pose of an aircraft from its 2D projected images.

Among the on-board vision methods, a binocular stereovision model established by Chen et al. [7] used stereo vision and the RANSAC (RANdom SAmple Consensus) algorithm to measure the pose of a non-cooperative target. Li et al. [8] used parallel binocular cameras to estimate the pose of a non-cooperative target based on stereo matching and 3D restructuring. Zhang et al. [9,10] proposed optimization-based methods to estimate the relative pose using stereo cameras, and the geometric structure of the non-cooperative target was exploited to improve the accuracy. Deng et al. [11] implemented an on-board binocular vision-based system to estimate the pose of Unmanned Aerial Vehicles (UAVs) for autonomous aerial refueling. Zhuang et al. [12] used the line features of the airport and the monocular camera on board to provide pose information for UAV autonomous landing. Benini et al. [13] estimated the pose of a UAV by detecting a marker composed of known circles for autonomous takeoff and landing. For scenes without known landmarks, the structure from motion (SFM) [14,15,16] method or the simultaneous localization and mapping (SLAM) [17,18] method can be leveraged to estimate the relative pose for aircraft navigation. A sequence of images is processed in these techniques, and a Kalman filter [19,20] is often used to reduce the pose error.

For external vision methods, the aircraft’s pose is often estimated using its 2D projected images captured by external imaging devices. Monocular cameras are widely used in external vison systems because the distance between aircraft and cameras is usually large. It is hard to estimate a 3D pose from a single 2D image without prior information such as 3D models of aircraft, synthetic aircraft image datasets, or acquired image sequences. Considering that pose estimation using complete aircraft models viewed from all aspects is storage- and time-consuming, feature extraction and pattern matching methods are proposed to reduce the dimension of pose estimation.

Hmam et al. [21] recognized aircraft based on a geometry-based reasoning system, and a generic model description of the aircraft was used for pose estimation. Wang et al. [22] combined a mathematical morphological algorithm and the Radon transform to extract the aircraft’s structure and used the average value of ordinary aircraft as a reference to calculate 3D pose parameters from 2D images. The use of a generic model of aircraft makes these algorithms more efficient and flexible, but this also leads to a reduction in the accuracy and robustness of pose estimation.

Breuers and Reus [23] used a Fourier-descriptor-based algorithm to estimate aircraft pose information. The method computes a Fourier descriptor to characterize the aircraft contour, and the pose information is estimated by comparing this Fourier descriptor to a reference database. Fu et al. [24] estimated the relative pose parameters of aircraft based on a contour model. The method first acquires 2D projections of a 3D model from different views and establishes a database; then, contour matching is employed to derive relative pose parameters. Wang et al. [25] estimated the pose of commercial aircraft in a runway end safety area using geometry structure features. This image-based method obtains aircraft pose information using the central moments of extracted geometry structure features and identifies an aircraft’s particular pose by a two-step feature matching strategy. Yuan et al. [26] proposed an aircraft pose recognition method based on locally linear embedding (LLE). In this method, LLE is applied for feature extraction and dimension reduction, and aircraft pose is recognized by propagation neural networks and nearest-neighbor algorithms. Although these methods reduce the complexity of the problem by feature extraction and pattern matching, 3D models of different aircraft are still needed, and a large amount of high-quality training data is necessary for pattern recognition to achieve accurate pose estimation, which reduces the flexibility and efficiency.

While there are a lot of features related to geometric structure to describe the pose information of aircraft, many of them were proposed for swept wing aircraft. Methods for straight wing aircraft structure extraction [27] and pose estimation are seldom addressed, despite the fact that the straight wing and its variants are the most common wing planform for low-speed aircraft [28]. With the rapid development of high-altitude long-endurance (HALE) UAVs, which often adopt a large-aspect-ratio straight wing design in order to increase lift [29,30], pose estimation of straight wing aircraft is of great importance.

In this article, we use a vision system located on the ground to estimate the pose of model-unknown straight wing aircraft. The vision system needs at least two monocular cameras to estimate the 3D pose. There are usually multiple cameras distributed in the flight test site or airport area, which allows our method’s requirements to be easily met. Compared to methods which rely on the use of 3D models and/or classifiers, only two 2D images obtained at the same time and some prior assumptions are explored in our approach to achieve accurate pose estimation for straight wing aircraft. We first identify the orientation of the fuselage and wings in an image pair using consistent line clustering; then, the planes intersection method is used to calculate the 3D pose information of the aircraft.

In the application scenario of this article, the dual-station photoelectric theodolite at a flight test site was used to estimate the absolute pose of an aircraft. The photoelectric theodolite tracks the aircraft, captures a sequence of images, and records the camera pose for every image frame. The image pair captured by the dual-station photoelectric theodolite was used to estimate the pose of the aircraft. To improve the measurement range and accuracy, two photoelectric theodolites with large baseline were selected and distributed on both sides of aircraft trajectory. Because of the large baseline and long-distance measurement, it is very difficult to obtain corresponding invariant features, and self-occlusion at certain angles would make feature matching more unreliable. In order to identify the main structure (fuselage and wings) of the aircraft in image pairs efficiently and robustly, the general geometry features of straight wing aircraft were analyzed.

In our method, line features extracted by the line segment detector (LSD) algorithm are used to describe the structure of the aircraft. The spatial and length consistency of line features is exploited to eliminate the disturbance of the background and unrelated parts of the aircraft, and parallel line segments are grouped into orientation-consistent clusters which represent the structure of the straight wing aircraft. To extract the aircraft’s structure accurately and robustly, a density-based clustering method is adopted according to the characteristics of the data. Mean shift and image moment methods are also used to improve the localization accuracy of the aircraft’s center in images. After recognizing the main structure of the straight wing aircraft in 2D images, the planes intersection method is used to determine the 3D pose. Our algorithm provides a universal framework to estimate the 3D pose of straight wing aircraft without relying on 3D models, cooperative markers, or other datasets.

The remainder of the paper is organized as follows: Section 2 introduces the coordinate system definition. Our pose estimation algorithm is explained in detail in Section 3. In Section 4, the experimental results of structure extraction and pose estimation are presented to validate our algorithm. Finally, Section 5 concludes this article.

## 2. Coordinate System Definition

**Figure 1:**
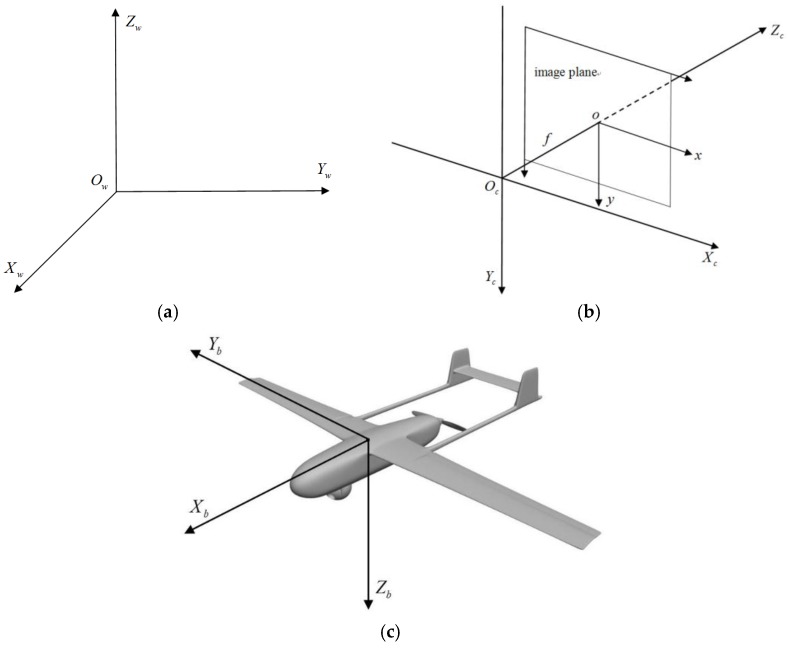
Coordinate systems: (**a**) World frame; (**b**) Camera frame; (**c**) Body frame.

In this section, we define several coordinate systems related to pose estimation. There are three major coordinate systems which are shown in Figure 1.

The world coordinate system (see Figure 1a) helps us track the aircraft and determine its position and attitude. We used the East-North-Up (ENU) coordinate system as the world frame.

The camera coordinate system, shown in Figure 1b, is attached to a camera which tracks the aircraft in the image plane. The origin of the camera frame is located at the optical center of the camera; the *x* axis is parallel to the horizontal axis of the image plane in the right direction, and the *z* axis is the optical axis of the camera in the right-handed coordinate system. Two cameras are used in our algorithm and are calibrated with respect to the world coordinate system; their poses are known for each image frame they record.

For the body coordinate system of straight wing aircraft shown in Figure 1c, the origin is located at the center of the aircraft. The *x* axis points along the fuselage reference line; the *y* axis is perpendicular to the fuselage plane of symmetry, directed to the right; and the *z* axis is perpendicular to the plane where the fuselage and wings are located in the right-handed coordinate system. For a straight wing or its variants, the wing edge lines are approximately parallel to each other, while line segments along the fuselage are approximately parallel to the fuselage reference line. Based on these geometry structure features, our pose estimation algorithm extracts the orientation of the fuselage reference line and the wing axis from which we can determine the orientation of the body coordinate axes of straight wing aircraft.

## 3. Pose Estimation Algorithm

The pose of an aircraft is represented by the transformation using a rotation matrix and translation vector which transform points in the body coordinate frame into points in the world coordinate frame. Our algorithm acquires the 3D pose information of an aircraft by determining the orientation of the body coordinate axes and the position of the body coordinate frame origin with respect to the world coordinate frame.

Our pose estimation algorithm first extracts the orientation of the fuselage and the wings in 2D image pairs, then uses plane–plane intersection to determine the 3D pose of the straight wing aircraft. The 2D pose information acquired by the structure extraction method is used as input to the planes intersection method to acquire the 3D pose of the aircraft. In the process of pose estimation, the initial pose information of the aircraft is needed to avoid ambiguity. In the following sections, the structure extraction and planes intersection methods will be explained in detail.

### 3.1. Structure Extraction Method

We propose a novel structure extraction method to identify the orientation of the fuselage and wings of straight wing aircraft in a 2D image without needing 3D models or other datasets. Due to the long-range imaging of the aircraft, reliable feature point correspondence is difficult to obtain, especially with ambiguities, extreme poses, or self-occlusions. To obtain the 2D pose information accurately and robustly, we use line features to describe the structure of the aircraft; line features are usually more accurate and robust than feature points in our application scenarios and also adapt to self-occlusions to some extent.

The geometric relations between line features are exploited to recognize the main structure of the aircraft. The most important geometric constraints used in our algorithm are the parallel constraints:

1. Line features distributed along the wing axis are approximately parallel to each other;
2. Line features along the fuselage reference line are approximately parallel to each other.

In addition, line features are concentrated in the area of the aircraft, and the lengths of line features on the main structure (fuselage and wings) of the aircraft are often larger than those on other parts of the aircraft. The main idea of our structure extraction method is to cluster line features based on these geometry constraints to acquire an accurate and robust estimation of the orientation of the aircraft’s main structure.

#### 3.1.1. Line Feature Extraction

**Figure 2:**
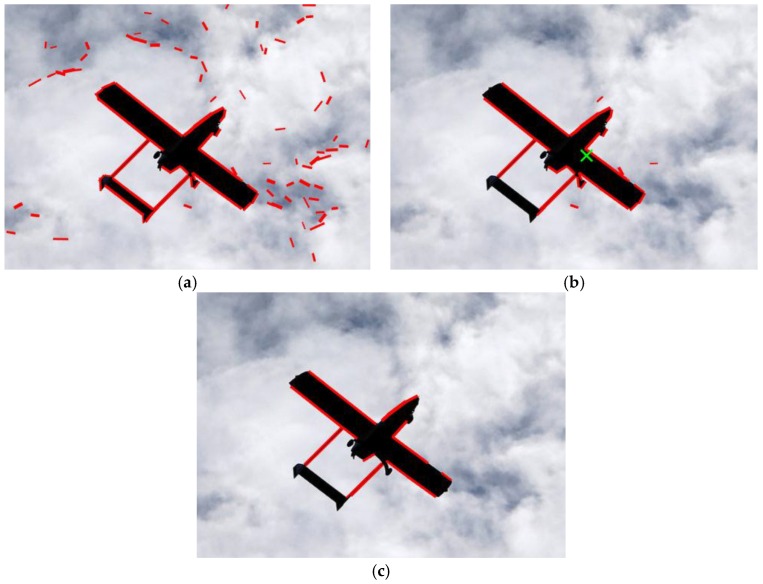
The results of line feature extraction and line clustering based on spatial and length consistency: (**a**) Line feature extraction; (**b**) Spatially consistent line clustering; (**c**) Length-consistent line clustering.

The state-of-the-art line segment detector (LSD) algorithm is utilized here to extract line features. The LSD algorithm, introduced by Gioi et al. [31], is a linear-time line segment detector giving results to subpixel accuracy, and a comparative study of line extraction methods by Zhang et al. [32] revealed that LSD is an optimal algorithm at different scales, blur degrees, and illumination. We detected 2D line segments using the LSD algorithm in an image to describe the structure of a straight wing aircraft. The result of the line feature extraction is shown in Figure 2a, where red line segments represent the detected line features. We denote the set of detected line segments as $S_{L}$.

#### 3.1.2. Spatially Consistent Line Clustering

Our algorithm is performed under the condition that the photographic image only contain one aircraft, which is common in actual application scenarios such as flight test, landing, or taking off. As the aircraft is a salient object in the image, detected line segments will be concentrated in the region of the aircraft and close to each other compared to irrelevant line segments, i.e., the density of line segments in the aircraft’s region is very high. Based on the location constraint of line segments, we performed spatially consistent line clustering to identify the center of the aircraft and rule out irrelevant line segments caused by the background.

We used the mean shift [33] algorithm to identify the center of the aircraft. Mean shift is a procedure for locating the maxima of a density function given discrete data sampled from that function. It is useful for detecting the modes of this density, which indicate the spatial consistency of line segments. The set of detected line segments $S_{L}$ was used as the input of the mean shift algorithm, and a Gaussian kernel K on the distance was used to determine the weight for re-estimation of the center. An image pixel x on a line segment which belongs to $S_{L}$ is represented by (x,y) where x and y are the horizontal and vertical coordinates of the pixel, respectively. The clustering center obtained by the mean shift algorithm is considered the aircraft’s center. The kernel function K and the weighted mean m(x) of the density can be represented as follows:

(1) $$\begin{array}{l} K(x_{i}-x)=e^{-c{\left\|x_{i}-x\right\|}^{2}}\\ m(x)=(\sum_{x_{i}\in n(x)}K(x_{i}-x)\cdot x_{i})\cdot {\left(\sum_{x_{i}\in n(x)}K(x_{i}-x)\right)}^{-1} \end{array}$$

where c is the weight of the kernel function and n(x) represents the neighborhood of point x. After determining the center of the aircraft, line segments within a certain distance of the clustering center are considered to belong to the aircraft, and other line segments are removed. Figure 2b shows the result of spatially consistent line clustering. The green cross in Figure 2b represents the cluster centroid of the mean shift, and red line segments indicate the reserved line features which are close to the estimated aircraft’s center. As we can see, many line segments which do not belong to the aircraft are rejected by spatially consistent line clustering.

The centroid of the aircraft can also be calculated via image moments, which is given by

(2) $${\tilde{x}}_{m}=\frac{\sum_{i=1}^{N}x_{i}}{N}\;{\tilde{y}}_{m}=\frac{\sum_{i=1}^{N}y_{i}}{N}.$$

**Figure 3:**
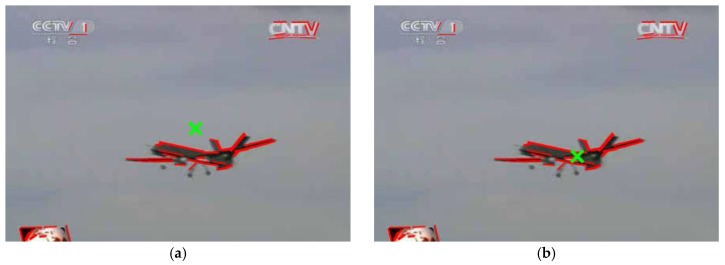
A comparison of the results of the methods: (**a**) Image moment method; (**b**) Mean shift algorithm.

Here, $\left({\tilde{x}}_{m},{\tilde{y}}_{m}\right)$ is the image coordinates of the aircraft’s center obtained by the image moment method, and N represents the number of pixels on the line segments. Although the image moment method can identify the centroid of $S_{L}$ without iteration, it is difficult to obtain the actual center of the aircraft robustly against a cluttered background. Figure 3 shows a comparison of the results of the image moment method and the mean shift algorithm, in which the estimated centroids of the aircraft are indicated by green crosses. The extracted 2D line features (red line segments in Figure 3) were used as the input of both methods. As we can see from Figure 3a, the result of the image moment method deviates from the actual center of the aircraft because of disturbance from the background, while the mean shift algorithm obtained a more accurate aircraft center and is partly resistant to a cluttered background. In the case of a cluttered background, the mean shift algorithm will be used to identify the center of the aircraft, and spatially consistent clustering provides an initial position estimation of the aircraft’s center which is then updated in the parallel line clustering.

#### 3.1.3. Length-Consistent Line Clustering

Although many noisy line segments are removed by spatially consistent line clustering, there are still some irrelevant line segments, as shown in Figure 2b. In this section, we use a length consistency criterion to further rule out irrelevant line segments. As the aircraft’s main structure (fuselage and wings) is usually larger than other parts such as tail or nose, line segments shorter than a certain threshold can be removed from the set of line segments. As the length of a line segment decreases, the uncertainty of its direction increases, i.e., a small position error of the endpoint causes greater direction error for shorter line segments. Excluding shorter line segments would improve the accuracy of 2D pose estimation in the following parallel line clustering.

The result of length-consistent line clustering is shown in Figure 2c. Compared to Figure 2b, the irrelevant line segments are excluded further, which is of benefit for the following clustering.

#### 3.1.4. Parallel Line Clustering

Parallel line clustering is the key step in the structure extraction algorithm and can acquire the directions of the fuselage and wings without relying on 3D models, other datasets, or cooperative markers. Line segments with similar directions are divided into one orientation-consistent line cluster. In the parallel line clustering process, the direction of a line segment is represented by the angle $\theta_{i}$ between the straight line that it belongs to and the horizontal axis of the image plane. The set of directions of line segments $\Theta =\left\{\theta_{1},\theta_{2},\ldots ,\theta_{N}\right\}$ is used as input to the parallel line clustering. The directions of the fuselage and the wings are denoted $\theta_{f}$ and $\theta_{w}$ respectively.

Weak perspective projection (scaled orthographic projection) is employed in parallel line clustering. As the size of the aircraft is small compared with its distance to the optical center along the optical axis, weak perspective approximation is valid. If line segments on the aircraft are parallel to each other in 3D space (the world frame), then this geometry feature of the corresponding line segments projected into the image plane remains unchanged under weak perspective projection.

For straight wing aircraft, line segments distributed along the wings are roughly parallel to each other, and angle values of these line segments are tightly concentrated around $\theta_{w}$ (small standard deviation), while angle values of line segments along the fuselage are concentrated around $\theta_{f}$. The directions of the fuselage and the wings can be extracted by clustering the high-density regions of Θ. According to the orientation feature of the line segments, a density-based clustering algorithm, density-based spatial clustering of application with noise (DBSCAN) [34], was used to group the parallel line segments into one orientation-consistent cluster containing the orientation information of the fuselage or the wings.

**Figure 4:**
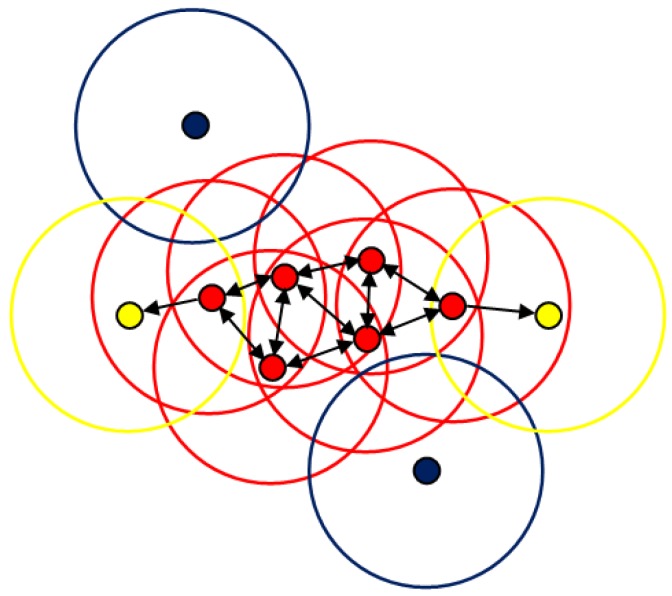
The explanation of the density-based spatial clustering of application with noise (DBSCAN) algorithm: the red points represent the core points, the yellow points represent the border points, and the blue points represent the noise points. The radius of the circle represents the distance threshold.

The data points used in DBSCAN clustering are the directions of line segments $\theta_{i}$. There are two parameters required to be specified in the DBSCAN algorithm, both of which are used to measure the density of data points. The first parameter is a distance threshold ε within which two data points close to each other will be grouped into one cluster. The distance threshold is the absolute difference between angle values in our algorithm. The second parameter is the minimum number of data points minPts needed to form an orientation-consistent cluster. Based on these two parameters, the data points are classified into three types, as shown in Figure 4:

- Core points: If a data point’s ε neighborhood contains at least minPts points, it is a core point (red points in Figure 4);
- Border points: If a data point’s ε neighborhood contains fewer than minPts points, but it is reachable from a certain core point (as indicated by one-way arrows in Figure 4), it is a border point (yellow points in Figure 4, the edge of a cluster);
- Noise points: If a data point is neither a core point nor a border point, it is a noise point (blue points in Figure 4).

The steps of the DBSCAN algorithm used for parallel line clustering are briefly described as follows:

1. For every data point $\theta_{i}$, search points in its ε neighborhood and use minPts to determine the core points in the set Θ.
2. Ignore all non-core points and group core points into parallel line clusters based on the connected components on the neighborhood graph (as indicated by two-way arrows in Figure 4).
3. For every non-core point, if it is in the ε neighborhood of a cluster, it is the border point of the cluster; otherwise, it is a noise point.

In contrast to traditional clustering methods such as *k*-means++ [35], the DBSCAN algorithm does not need to specify the number of clusters in advance and is robust to outliers. It forms clusters based solely on the spatial density of the data. The two orientation-consistent clusters with minimum interclass variance represent the structure of the fuselage and the wings which contain the orientation information of the aircraft in the image. The directions of the fuselage and the wings are obtained by extracting the centers of the parallel line clusters.

In our method, the directions of the fuselage and the wings are distinguished based on an initial pose constraint. The approximate orientation of the aircraft needs to be specified in the initial frame of the image sequence to avoid ambiguity and to help identify the actual pose of the aircraft. With this condition, the directions of the fuselage and the wings can be distinguished in the initial frame, and the orientation information of the current frame will be used in the next frame. In the application scenarios of our algorithm, such as take-off, landing, or flight testing, this condition is easily met. In practice, the pitch angle (or the yaw angle) and the roll angle of the aircraft are provided, or the approximate positions of the nose and one wing tip are marked in one image of the initial image pair.

After obtaining the orientation-consistent clusters, irrelevant line segments are removed from the set $S_{L}$, and the position of the aircraft’s center is then re-estimated from the set $S_{L}$ based on the image moment method. Since only line segments on the main structure of the aircraft are left, it is possible to identify the center of the aircraft with higher precision.

**Figure 5:**
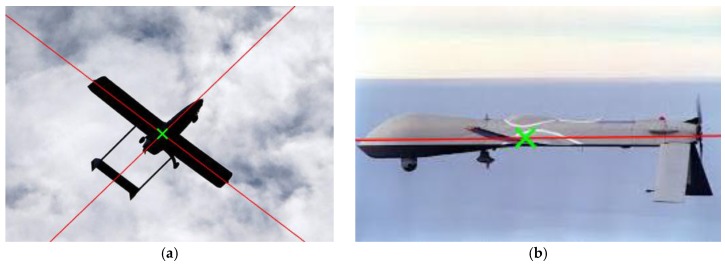
The results of parallel line clustering. (**a**) The directions of the fuselage and the wings are extracted correctly; (**b**) The direction of the wings cannot be extracted for this extreme pose.

The results of parallel line clustering are shown in Figure 5. As shown in Figure 5a, the red straight lines indicate the directions of the fuselage and the wings, and the green cross indicates the estimated centroid of the aircraft. The directions of the fuselage and the wings were correctly extracted by parallel line clustering. However, in Figure 5b, there is only one cluster with enough parallel line segments for this extreme pose. In this case, the direction of only the fuselage or the wings can be acquired by parallel line clustering, and the unknown direction needed for pose estimation is replaced by the corresponding orientation information of the previous frame.

### 3.2. Planes Intersection Method

After the structure extraction method determines the pixel coordinates of the aircraft’s center and the directions of the fuselage and the wings in an image pair, the planes intersection method is used to estimate the 3D pose of the aircraft.

Two cameras were used in the intersection measurement and are indicated by their projection matrices $P_{1}$ and $P_{2}$. The camera projection matrices are of the form

(3) $$P_{1}=K_{1}\left[R_{1}|t_{1}\right]\;\;P_{2}=K_{2}\left[R_{2}|t_{2}\right]$$

where $K_{i}\;(i=1,2)$ is the camera intrinsic matrix of the camera, and $R_{i}$ and $t_{i}$ represent the rotation and translation, respectively, of the corresponding camera with respect to the world frame. We assume that the cameras are calibrated with respect to the world frame and that the $P_{i}$ are known.

The camera model is represented as

(4) $$zx=P_{i}X$$

where $X={\left(X,Y,Z,1\right)}^{T}$ is the world coordinates and $x={\left(u,v,1\right)}^{T}$ is the image coordinates of X. As weak perspective projection is employed, z is a positive constant.

The image pair captured by the two cameras at the same time is denoted $\langle I_{1},I_{2}\rangle$. The center of the aircraft obtained by the structure extraction method in the image $I_{i}\;(i=1,2)$ is represented as $AC_{i}=(x_{i},y_{i})$ where $x_{i}$ and $y_{i}$ are the horizontal and vertical coordinates of the image, and the directions of the fuselage and the wings are represented as $\theta_{i}^{f}$ and $\theta_{i}^{w}$, respectively.

**Figure 6:**
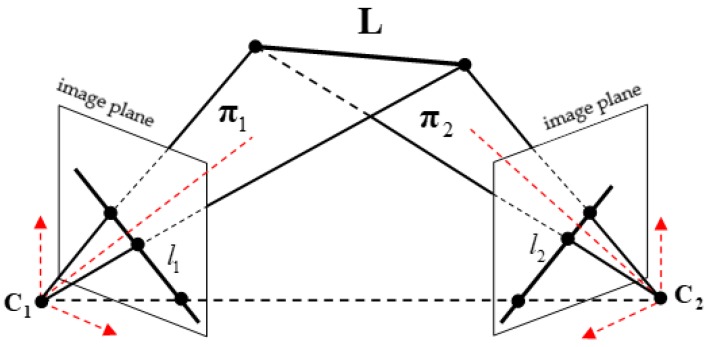
Geometric constraint of plane–plane intersection.

Figure 6 explains the geometric constraint of the planes intersection method. As shown in Figure 6, the two cameras are indicated by their optical centers $C_{1}$ and $C_{2}$ and by image planes. The 3D line in the world coordinate system is represented as L, which is the line of intersection of the two planes $\pi_{1}$ and $\pi_{2}$; $l_{i}\;(i=1,2)$ is the projected line of L in the image plane; and the plane $\pi_{i}$ is determined by the line L and the optical center $C_{i}$. Let the normalized vector V of L represent the direction of the fuselage or the wings; the planes intersection method estimates the 3D attitude of the aircraft by obtaining the solution of V.

The projected line $l_{i}$ in the image plane is identified by $AC_{i}$ and $\theta_{i}^{f}$ (or $\theta_{i}^{w}$); an analytical expression of $l_{i}$ is

(5) $$a_{i}u+b_{i}v+c_{i}=0.$$

Equation (5) can be represented in vector form as the following:

(6) $$\left[\begin{array}{ccc} a_{i} & b_{i} & c_{i} \end{array}\right]x=0.$$

By substituting Equation (6) into Equation (4) for each camera, we obtain

(7) $$z\left[\begin{array}{ccc} a_{i} & b_{i} & c_{i} \end{array}\right]x=\left[\begin{array}{ccc} a_{i} & b_{i} & c_{i} \end{array}\right]P_{i}X=0,$$

and the plane $\pi_{i}$ can be expressed as

(8) $$\begin{array}{l} {}[{\begin{array}{cccc} A_{i} & B_{i} & C_{i} & D \end{array}}_{i}]X=0\\ {\vec{n}}_{i}=\left[\begin{array}{ccc} A_{i} & B_{i} & C_{i} \end{array}\right] \end{array}$$

where ${\vec{n}}_{i}$ is the normalized vector of the plane $\pi_{i}$. Note that Equations (7) and (8) have the same form, and $\left[\begin{array}{ccc} a_{i} & b_{i} & c_{i} \end{array}\right]P_{i}$ is already known; the normalized vector ${\vec{n}}_{i}$ is derived from Equations (7) and (8). After we obtain the normalized vectors ${\vec{n}}_{1}$ and ${\vec{n}}_{2}$, the normalized vector V which contains the orientation information of the aircraft is solved as follows:

(9) $$V={\vec{n}}_{1}\times {\vec{n}}_{2}.$$

As the 3D line L can be parametrized in the world coordinate frame by the two planes $\pi_{1}$ and $\pi_{2}$ as a 2 × 4 matrix, let $L_{f}$ be the 3D line parallel to the fuselage reference line and $L_{w}$ be the 3D line parallel to the wing edge lines. The point of intersection of $L_{f}$ and $L_{w}$ is the center of the aircraft. By calculating the respective normalized vectors of $L_{f}$ and $L_{w}$ using Equation (9), we can obtain the 3D attitude of the straight wing aircraft. The rotation matrix is calculated by singular value decomposition, and the initial pose constraint is used to avoid reflective ambiguity. In order to obtain the world coordinates of the point of intersection of $L_{f}$ and $L_{w}$, which determine the 3D position of the aircraft, overdetermined equations are established as follows.

(10) $$\begin{array}{l} AX=0\\ A=\left[\begin{array}{c} L_{f}\\ L_{w} \end{array}\right] \end{array}$$

Here, A is a 4 × 4 matrix and X represents the point of intersection of the two lines (the translation vector). The overdetermined equations AX=0 can be solved by singular value decomposition, and the solution is the singular vector corresponding to the smallest singular value of A. Before solving the overdetermined equations, an optimal estimator for the center point based on the epipolar constraint can be used to reduce the geometric error [36].

The 3D attitude of the aircraft is determined by the normalized vectors of $L_{f}$ and $L_{w}$, and the 3D position of the aircraft is determined by the point of intersection X of the two lines. As we assume that $L_{f}$ and $L_{w}$ are coplanar in our pose estimation algorithm, the ambiguity will occur during the process of pose estimation, and the initial pose constraint will be used to determine the unique solution. Based on the results of the structure extraction method, the planes intersection method can acquire the 3D pose of the straight wing aircraft. Moreover, our pose estimation algorithm can easily be extended to multiple camera views.

### 3.3. Algorithm Summary

In this section, we summarize the whole pose estimation algorithm as is shown in Algorithm 1.

**Algorithm 1:** Pose estimation based on consistent line clustering and planes intersectionInput:The image pair $\langle I_{1},I_{2}\rangle$, the two camera matrices $P_{1}$, $P_{2}$, and the initial pose constraint.Output:The 3D position and 3D attitude of the straight wing aircraft.Step 1Extract line features in image pairs using the LSD algorithm;Step 2Locate the center of the aircraft in the 2D images and cluster spatially consistent line segments;Step 3Rule out line segments shorter than a certain threshold;Step 4Classify line segments into orientation-consistent clusters, extract the directions of the fuselage and the wings in the image pair, and re-estimate the center of the aircraft;Step 5Calculate the 3D attitude and 3D location using the plane–plane intersection method.

**Figure 7:**
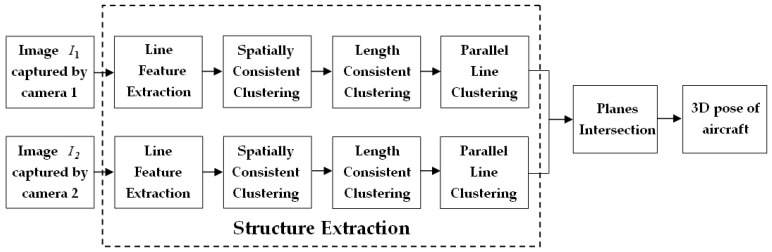
The flowchart of our pose estimation algorithm.

The flowchart of the algorithm is shown in Figure 7.

## 4. Experiments and Results

Experiments were performed to validate the effectiveness and accuracy of the proposed structure extraction and pose estimation methods. Real images of different straight wing aircraft downloaded from the Internet were used to demonstrate the effectiveness and universality of the structure extraction method, and simulated images of straight wing aircraft were exploited to evaluate the accuracy of our pose estimation algorithm. Our method was implemented using MATLAB on a laptop equipped with an Intel Core i7 CPU with a 2.80 GHz processor and 8.00 GB of RAM.

### 4.1. Experimental Results of Structure Extraction

The qualitative evaluation of our structure extraction method was performed using real images downloaded from the Internet. A total of 60 images of different sizes were downloaded and used in the experiment. Each image contains one aircraft whose planform is the straight wing or its variant, and the structure extraction method was used to identify the orientation of the aircraft’s main structure in a single 2D image. Among these images, some are challenging for structure extraction since they contain a cluttered background, other objects, random noise, or perspective effects.

**Figure 8:**
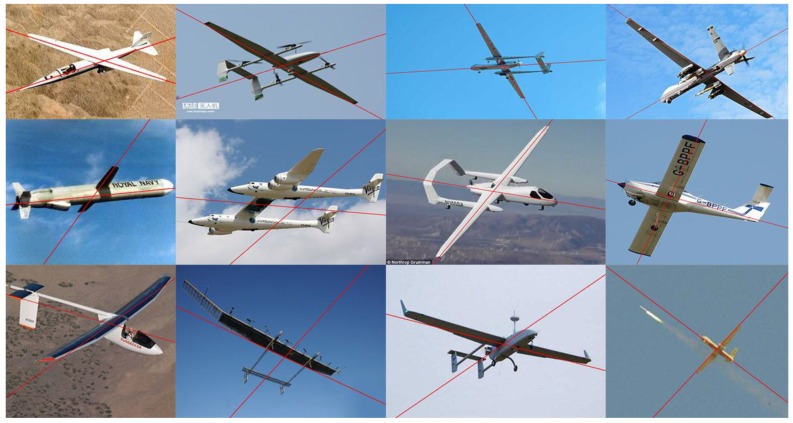
Results of the structure extraction method.

In the experiment, the directions of the fuselage and the wings in 51 of the 60 images were correctly identified. Figure 8 shows some of the results of structure extraction. As we can see, our structure extraction algorithm can be applied flexibly to different types of straight wing aircraft without needing 3D models of aircraft or other datasets, and it can also deal with different aircraft poses effectively and robustly extract the main structure under self-occlusion or a cluttered background. Moreover, the parallel assumption does not need to hold strictly. Even if perspective effects exist or the line segments are not strictly parallel to each other, our algorithm can still recognize the main structure of the aircraft.

**Figure 9:**
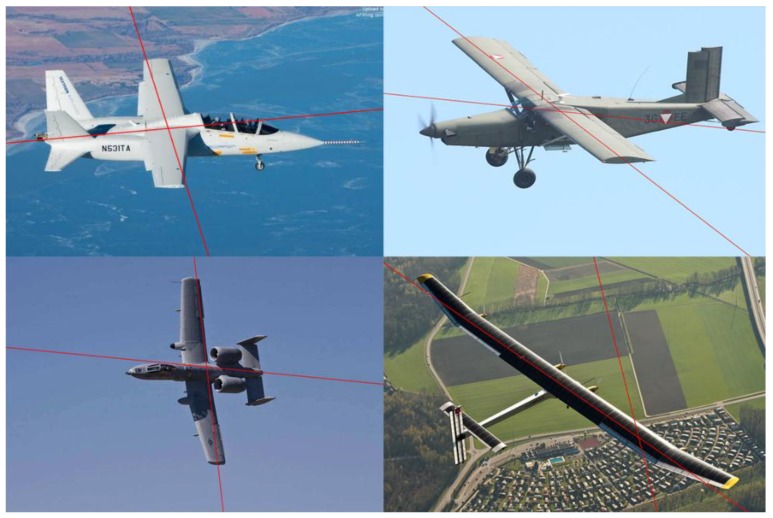
Some incorrect results from our structure extraction algorithm.

While the algorithm achieved good results in most downloaded images, Figure 9 shows some cases in which our structure extraction method obtained incorrect results. There are two main reasons for these incorrect results:

1. The structure of the aircraft (fuselage or wings) does not satisfy the assumption of parallel line clustering, i.e., the line segments distributed along this structure are not parallel to each other in the image (see row 1, Figure 9).
2. Some parts of the aircraft (tail or external mounts) or the background affect the consistent line clustering (see row 2, Figure 9).

Changing weather or light conditions may also affect the success rate of our algorithm. When the weather condition or brightness/darkness level changes, the edges of the aircraft’s main structure may be blurred during image acquisition, and unreliable line features will be detected. Changing weather or light conditions may affect the accuracy and robustness of the line feature detection, which in turn disturbs the consistent line clustering results and reduces the accuracy and success rate of our algorithm. In our method, the LSD algorithm used to detect line features can adapt to optical blur and illumination changes to some extent.

The situations shown in Figure 9 are uncommon in our application scenarios, and despite the fact that our algorithm is mainly for estimating the pose of a straight wing aircraft at long distance, the experimental results show that the proposed algorithm is able to recognize the aircraft’s main structure robustly even at close range.

### 4.2. Experimental Results of Pose Estimation

**Figure 10:**
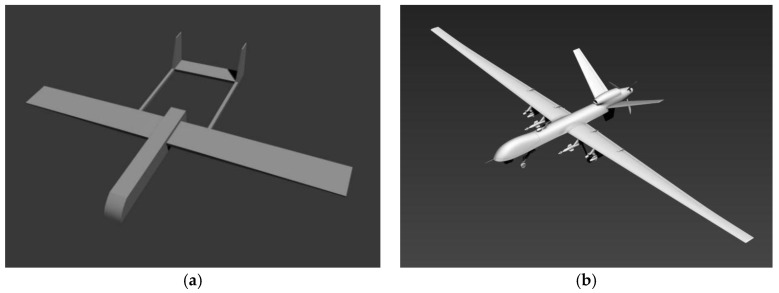
Two aircraft models: (**a**) Model 1; (**b**) Model 2.

Simulated image pairs were used to test our pose estimation algorithm. Two models were used in our experiment to simulate straight wing aircraft, as shown in Figure 10. These two models were created using Autodesk 3ds Max [37], which is a professional 3D computer graphics program for making 3D animations, models, and images. Model 1 (see Figure 10a) represents a general commercial UAV with standard straight wings while Model 2 (see Figure 10b) is a full-size simulation of the MQ-9 unmanned aircraft which has straight tapered wings (a variant of the standard straight wing). The size of Model 1 is 3.4 m × 5.0 m × 0.7 m (length, width, height), and the size of Model 2 is 10.4 m × 24.8 m × 3.1 m (length, width, height).

Two cameras in 3ds Max were used to simulate the dual-station photoelectric theodolite at the flight test site. The internal parameters and spatial layouts of the cameras for aircraft pose estimation are shown in Table 1. As we can see from Table 1, flight simulation scenarios were established for Model 1 (Scene 1, see Table 1) and Model 2 (Scene 1, see Table 1).

In our simulation experiments, cameras with different internal parameters and spatial layouts were used to test the performance of our algorithm, and the two cameras in the scene were located on both sides of the aircraft trajectory. The location coordinates of the cameras were in the East-North-Up (ENU) coordinate system. In Scene 1, the baseline between the two cameras was 55.23 m, while the two cameras in Scene 2 had a baseline of 957.55 m. The image pairs were generated by the two cameras in the scenes, and it is very difficult to obtain reliable feature correspondences in these wide-baseline images.

In order to test the performance of our pose estimation algorithm on different poses in simulation image pairs, we rotated Model 1 around the x, y, and z axes to simulate changes in the roll angle γ, pitch angle ψ, and yaw angle φ, respectively. Table 2 shows the selected rotation angles $\left(\theta_{x},\theta_{y},\theta_{z}\right)$ of Model 1, where $\theta_{x}$ represents the roll angle, $\theta_{y}$ represents the pitch angle, and $\theta_{z}$ represents the yaw angle. As detailed in Table 2, 13 image pairs were generated for Model 1, and the selected angle range was reasonable considering actual flight situations. The translation vector of Model 1 in Scene 1 was $T_{true}=(0,0,20\;m)$.

For Model 2 in Scene 2, an aircraft trajectory was designed to simulate the flight. During the flight simulation, the pitch angle of Model 2 varied from −15° to 15°, the roll angle varied from −10° to 10°, and the translation vector was $T_{true}=(x,0,200\;m)$, where x ranged from 0 to 600 m. The simulated flight path was rendered into 13 image pairs in steps of 50 m in Scene 2, and the rotation angles of each step are shown in Table 3.

**Figure 11:**
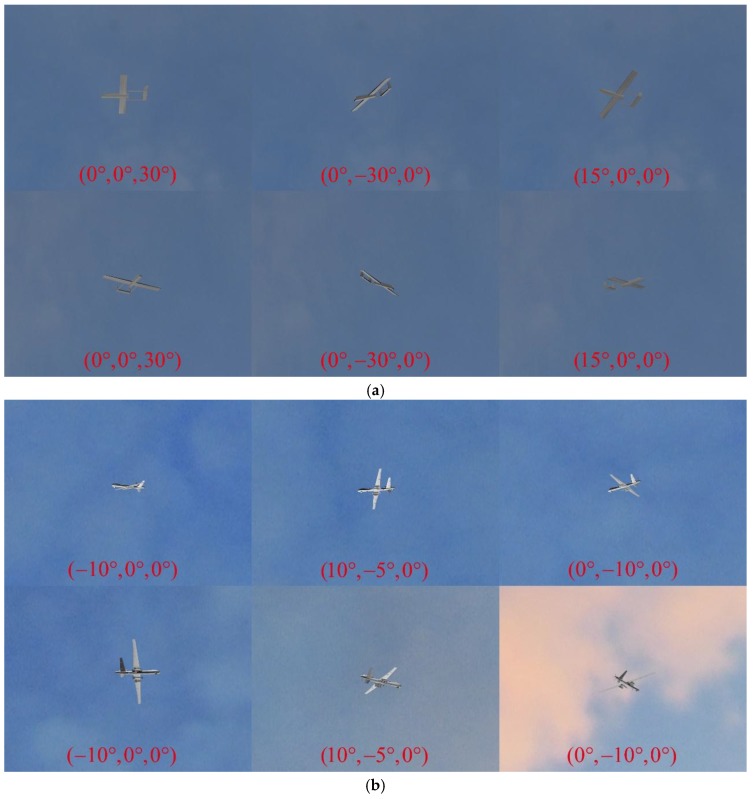
Examples of simulated image pairs generated by the 3ds Max rendering engine: (**a**) Image pairs of Model 1; (**b**) Image pairs of Model 2.

We used the 3ds Max rendering engine to generate the simulated image pairs of these two models; these are shown in Figure 11a,b. In Figure 11a, the top row and the bottom row represent the simulated images of Model 1 captured by Camera 1 and Camera 2, respectively, in Scene 1, and every column represents an image pair captured at the same time. In Figure 11b, the top row and the bottom row represent the simulated images of Model 2 captured by Camera 1 and Camera 2, respectively, in Scene 2, and every column represents an image pair captured at the same time. The rotation angles $\left(\theta_{x},\theta_{y},\theta_{z}\right)$ of the aircraft in each shot are also displayed in Figure 11.

In order to make the simulation scenes more realistic, a sky background with clouds and different types of natural light was also simulated (see Figure 11). As shown in Figure 11, the wide-baseline image pairs contain aircraft with different scales, poses, and self-occlusion, and optical blur exists due to long-range imaging. Under these challenging circumstances, a robust algorithm is needed to obtain accurate pose information from a single image pair.

**Figure 12:**
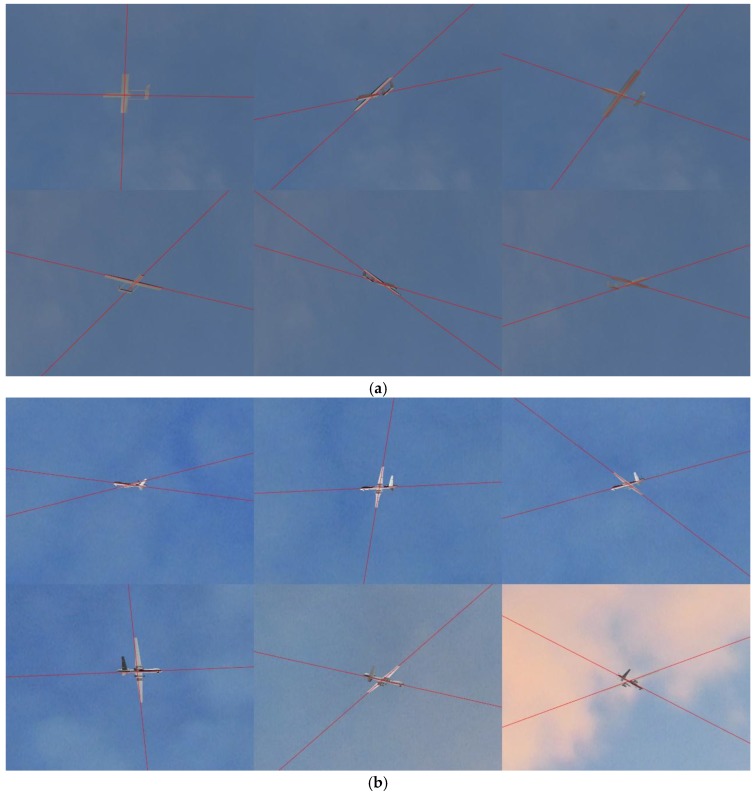
Structure extraction results on the simulated images: (**a**) Results on Model 1’s simulated image pairs; (**b**) Results on Model 2’s simulated image pairs.

Figure 12 presents the results of our structure extraction method on the simulated image pairs shown in Figure 11. The directions of the fuselage and the wings are indicated by the red lines in Figure 12. As we can see, the main structure of the aircraft was correctly extracted by our structure extraction method, and the results further validate the performance of our method. The 3D pose of the aircraft can be obtained effectively only when the 2D pose information in the image pair is extracted robustly and accurately.

We compared our pose estimation algorithm with Li’s method [8] and pose estimation errors were used to evaluate the algorithms. In Li’s method, the 3D pose of a non-cooperative target is estimated by a stereo camera based on a triangulation method, and the feature points obtained by the line feature extraction are used for stereo matching and 3D reconstruction. The triangulation method is typically applied to estimate 3D position in computer vision, and the pose estimation pipeline of Li’s method is also widely used, so it was selected for comparison to validate our proposed method.

For the ground truth pose of the aircraft ($R_{true}$ and $T_{true}$) and corresponding estimated pose ($\hat{R}$ and $\hat{T}$), the rotation error is calculated by $error_{rot}=\left\|\hat{\theta }-\theta_{true}\right\|$ where $\hat{\theta }$ and $\theta_{true}$ are the Euler angles of $\hat{R}$ and $R_{true}$, respectively, and the translation error is calculated by $error_{trans}=\left\|\hat{T}-T_{true}\right\|$.

Since Li’s method can hardly obtain reliable feature matching results across these wide-baseline views in our experiments, we manually removed mismatched features, selected correct matches in the image pairs, and confirmed that there were enough corresponding feature points for pose estimation. The 3D models are also used in Li’s method to obtain the absolute pose of the aircraft, while our algorithm is model free and acquires the 3D pose information automatically.

**Figure 13:**
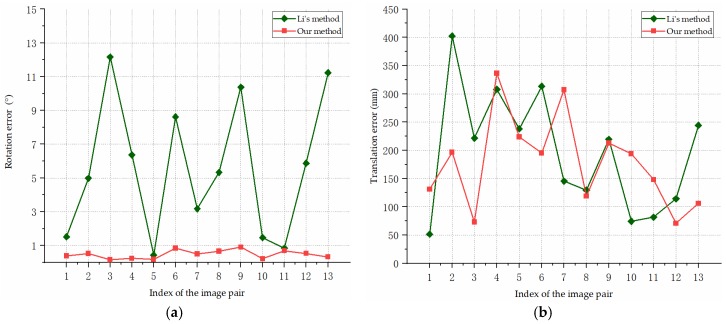
Pose estimation errors for the simulated images of Model 1: (**a**) Rotation errors; (**b**) Translation errors.

Figure 13 shows the pose estimation errors of our algorithm and Li’s method for the simulated images of Model 1. In Figure 13a, the rotation errors are presented, and the translation errors are shown in Figure 13b. As we can see from Figure 13, the translation accuracy of our method is similar to that of Li’s method, and our pose estimation method performs consistently better than the compared method in the estimation of the rotation angle. The rotation angle errors of our method are within 1°, the average rotation error is 0.47°, and the average translation error is 177.91 mm.

**Figure 14:**
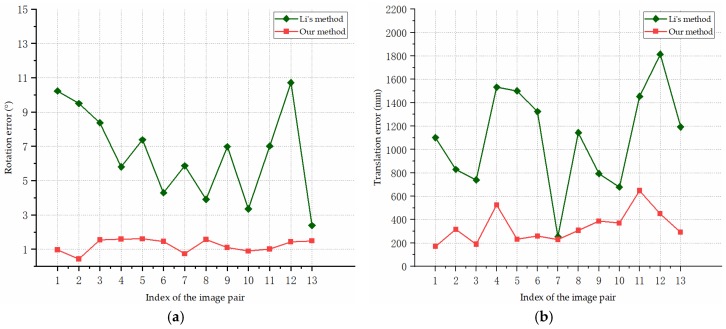
Pose estimation errors for the simulated images of Model 2: (**a**) Rotation errors; (**b**) Translation errors.

Figure 14 shows the pose estimation errors of our algorithm and the compared method for the simulated images of Model 2. In Figure 14a, the rotation errors are presented, and the translation errors are shown in Figure 14b. Model 2 is more complex than Model 1 and there is a greater imaging distance in Scene 2, while our algorithm still achieves accurate and stable pose estimation results compared to the results for Model 1. In Figure 14, the proposed method outperforms the compared method in the estimation of the rotation angle and translation vector, which is due to the accuracy and robustness of our structure extraction and planes intersection methods. The large fluctuations in the result curves indicate that the triangulation process used in Li’s method is sensitive to various errors. The triangulation method uses the intersection of two lines to estimate the 3D position; with the measure distance increasing, the uncertainty increases, making the results more sensitive to noise. The average rotation error of our method is 1.21°, and the average translation error is 336.49 mm. The experimental results indicate that our method can extract the structure and estimate the pose accurately.

In addition, our method is also efficient. We ran our method 1000 times and recorded the execution time. The average execution time was 30.74 ms (including the structure extraction and planes intersection methods), which means that our algorithm can estimate the 3D pose efficiently.

The simulation experiment results show that our algorithm estimates the pose of the straight wing aircraft more accurately and robustly than does the compared method. Meanwhile, our method is efficient and flexible and can be applied to different types of straight wing aircraft.

## 5. Conclusions

An accurate and robust pose estimation method for straight wing aircraft was proposed in this paper. The geometry structure features of straight wing aircraft were utilized for structure extraction and the pose information was acquired by the planes intersection method. Our method establishes a universal framework for pose estimation of straight wing aircraft without relying on 3D models or other datasets, unlike other existing methods, and can be extended to other targets with similar geometric constraints. For an aircraft without similar geometric constraints to straight wing aircraft, our proposed method is unable to extract its main structure robustly and accurately. In the case of a swept wing aircraft, only the fuselage contains enough parallel lines can be detected effectively, while the wings cannot be extracted accurately. Extending our algorithm to aircraft with different wing planforms will be the focus of our future research.

Our method can also provide initial pose information for algorithms with higher precision efficiently. For an image sequence captured during flight, our future work will also focus on using an extended Kalman filter or particle filter to improve the accuracy of our algorithm.

## Figures and Tables

**Table 1:** The internal parameters and spatial layouts of the cameras in Scene 1 and Scene 2.

	Camera	Focal Length	Field of View	Image Resolution	Location (x,y,z)
Scene 1	1	70 mm	28.842°×21.832°	1280×960	(−15 m,−25 m,0)
	2	75 mm	26.991°×20.408°	1280×960	(−20 m,30 m,0)
Scene 2	1	300 mm	6.867°×5.153°	1280×960	(350 m,550 m,30 m)
	2	275 mm	7.49°×5.621°	1280×960	(170 m,−390 m,0)

**Table 2:** The selected rotation angles of Model 1 in Scene 1.

	1	2	3	4	5	6	7	8	9	10	11	12	13
$\theta_{x}$	0°	0°	0°	0°	0°	0°	0°	0°	0°	0°	0°	−15°	15°
$\theta_{y}$	0°	0°	0°	0°	0°	0°	0°	−30°	30°	−15°	15°	0°	0°
$\theta_{z}$	0°	−30°	30°	−60°	60°	−90°	90°	0°	0°	0°	0°	0°	0°

**Table 3:** The rotation angles of Model 2 in Scene 2.

	1	2	3	4	5	6	7	8	9	10	11	12	13
$\theta_{x}$	0°	0°	0°	−10°	−5°	−5°	10°	0°	5°	5°	0°	0°	0°
$\theta_{y}$	0°	0°	0°	0°	15°	10°	−5°	5°	−5°	0°	−15°	−10°	0°
$\theta_{z}$	0°	0°	0°	0°	0°	0°	0°	0°	0°	0°	0°	0°	0°
